# Development of a set of core outcome measures for ambulant children with cerebral palsy after lower limb orthopaedic surgery

**DOI:** 10.1111/dmcn.70133

**Published:** 2025-12-29

**Authors:** Hajar Almoajil, Sally Hopewell, Helen Dawes, Francine Toye, Rakhshan Kamran, Tim Theologis

**Affiliations:** ^1^ Department of Physical Therapy College of Applied Medical Science, Imam Abdulrahman Bin Faisal University Al Khobar Saudi Arabia; ^2^ Nuffield Department of Orthopaedics, Rheumatology and Musculoskeletal Sciences University of Oxford Oxford UK; ^3^ College of Medicine and Health, University of Exeter Exeter UK; ^4^ Nuffield Orthopaedic Centre, Oxford University Hospitals NHS Foundation Trust Oxford UK; ^5^ Department of Medical Imaging University of Toronto Toronto Ontario Canada

## Abstract

**Aim:**

To develop consensus on a core set of standardized outcome measures to be applied to each domain of the previously developed core outcome set for lower limb orthopaedic surgery for ambulant children with cerebral palsy (CP).

**Method:**

This work consisted of the following three steps: (1) a scoping review of the literature to identify previously used outcome measures to assess lower limb orthopaedic surgery of ambulant children with CP; (2) searching the COnsensus‐based Standards for the selection of health Measurement Instruments (COSMIN) and PubMed databases to assess the quality of the psychometric properties of outcome measures and feasibility criteria; and (3) a consensus meeting with seven healthcare professionals with expertise in CP research and in the assessment of outcome measure psychometric properties was held in September 2021. Consensus on the outcome measures core set was developed through presentation of the evidence and whole‐group discussions.

**Results:**

A combination of clinician‐driven and patient‐reported outcome measures was considered the most appropriate way to assess the outcome of orthopaedic surgical interventions. Agreement was reached on seven core outcome measures: three‐dimensional gait analysis, Edinburgh Visual Gait Scale, Gross Motor Function Measure, Gait Outcome Assessment List, Gillette Functional Assessment Questionnaire, Patient‐Reported Outcome Measure Instrument System (pain interference, and fatigue), and Cerebral Palsy Quality of Life for Children questionnaire.

**Interpretation:**

This study recommends a set of core outcome measures for use in research on lower limb orthopaedic surgery for ambulant children with CP. Consistent use of this core set would enhance validity and comparability of future research.

## Abstract 2

**This original article is commented on by Boyer on pages** 1027‐1028 **of this issue.**

**Abbreviations**
COMET: Core Outcome Measures in Effectiveness Trials
COSMIN: COnsensus‐based Standards for the selection of health Measurement INstruments
CP‐QoL: Cerebral Palsy Quality of Life for Children
EVGS: Edinburgh Visual Gait Scale
Gillette FAQ: Gillette Functional Assessment Questionnaire
GMFM‐66: Gross Motor Function Measure
GOAL: Gait Outcome Assessment List
ICF: International Classification of Functioning, Disability and Health
PROMIS: Patient‐Reported Outcomes Measurement Information System
QoL: quality of life
3DGA: three‐dimensional gait analysis

**What this paper adds**

- Consensus was reached on seven outcome measures to support comparability and implementation.
- Adoption of the core measures will improve research consistency.
- Adoption will also improve quality of lower limb orthopaedic surgery for ambulant children with cerebral palsy.
- This core set of outcome measures provides a robust foundation for conducting meta‐analyses within the field of evidence‐based medicine.

Outcome measures provide a common language among researchers and clinicians in determining the success and impact of an intervention.^1^, ^2^ Previous literature reviews have demonstrated significant variability in the outcome measures used after lower limb orthopaedic surgery to improve gait in children with ambulant cerebral palsy (CP).^3^, ^4^ With a growing demand for orthopaedic surgical interventions for children with CP, outcome measures are necessary tools to assess clinical effectiveness in this field.^5^ Outcome measures need to be valid, reliable, and responsive to change, otherwise there is a risk of imprecise or biased results.

Most studies on lower limb surgery report results in terms of CP impairment (e.g. spasticity, joint range of motion, gait pathology) using objective outcome measures.^4^, ^6^, ^7^, ^8^ However, it is becoming clear that the success of a procedure is not merely judged by the alleviation of any symptoms or deformity correction, but also determined by its impact on the child's life (e.g. daily life activities, social participation, quality of life [QoL]).^9^ There has been a shift of the emphasis away from objective outcome measures towards self‐ or proxy‐reported measures of surgical outcomes.^3^ This has led to an increase in the development of patient‐reported outcome measures designed to assess the impact of the surgery from the patients' and/or carers' perspectives.^10^

Given that the number of available outcome measures has significantly increased, deciding which outcome measures to use is becoming a challenge.^11^ It is not surprising that there is inconsistency and heterogeneity in outcome measures across studies in this field. There are multiple outcome measures available for each health construct (outcome domain).^3^, ^4^ For example, a review published in 2020 identified 29 outcome measures used in research between 2016 and 2019 for the measurement of lower limb orthopaedic surgical outcomes in ambulant children with CP.^3^ This variability increases the risk of poor‐quality outcome measures being used, which can introduce information bias into research and practice. Another problem is that the inconsistency and variability of outcome measures hampers the comparability of results and makes conducting meta‐analysis challenging.^3^, ^4^, ^12^

The problems arising from the inconsistent use of outcome measures can be addressed by the development of a core outcome set, as proposed by the Core Outcome Measures in Effectiveness Trials (COMET) initiative.^13^, ^14^ A core outcome set is an agreed minimum set of outcomes to be measured and reported in all clinical research and practice in a specific health condition or intervention. The development of a core outcome set is a two‐step process: first, determining which outcome domains should be included in the core outcome set; and second, achieving consensus on the most appropriate outcome measures, the tools to be used for these outcomes to be measured.^14^, ^15^

The development of a core outcome set of domains to be used after lower limb orthopaedic surgery in children with CP has been previously completed with involvement of children and young adults with CP, representatives (family members or carers), and health professionals (clinicians and researchers).^3^, ^16^, ^17^, ^18^ Consensus was achieved on 19 core outcomes in eight domains: pain and fatigue, lower limb structure, motor function, mobility (daily life activities), gait‐related outcomes, physical activity, independence, and QoL.^16^

The aim of this current study was to develop recommendations for appropriate clinical and patient‐reported outcome measures to be used in assessing the above outcome domains. The COnsensus‐based Standards for the selection of health Measurement INstruments (COSMIN) initiative (http://www.cosmin.nl/) was used for the optimal selection of outcome measures. The objectives of this study were (1) to determine the quality and feasibility (ease‐of‐use) of the most frequently used outcome measures in the field of lower limb orthopaedic surgery for ambulant children with CP and (2) to formulate consensus‐based recommendations on a set of core outcome measures to cover the previously defined eight core outcome domains.^16^

## METHOD

The research methods are based on published guidelines developed by COMET and COSMIN in 2016.^19^ A study protocol defining objectives and the consensus methodology for this study have been published previously.^12^ The selection of core outcome measures comprises the following steps: (1) conceptual considerations (i.e. scope); (2) identification of existing outcome measures; (3) quality assessment of outcome measures; and (4) generic recommendations on the selection of outcome measures for a core measures set. Figure S1 summarizes the method used to develop this standard set of outcome measures.

### Step one: conceptual considerations

The aim of this step was to define the target population and construct (i.e. outcome or domain) to be measured. The target population included ambulant children and young people aged between 6 years and 18 years, classified in levels I, II, or III of the Gross Motor Function Classification System (GMFCS).^20^ The core outcome measures would aim to cover the previously identified core domains discussed above: (1) pain and fatigue, (2) lower limb structure, (3) motor function, (4) mobility (daily life activities), (5) gait‐related outcomes, (6) physical activity, (7) independence, and (8) QoL.^16^

### Step two: identify existing outcome measures

To identify outcome measures, a scoping review was conducted on the basis of our previous review of the existing literature on lower limb orthopaedic surgery for ambulant children with CP.^3^ A systematic search (from 1st January 2016–30th July 2019) was also performed in five databases: PubMed, CINAHL, MEDLINE, Embase, and Cochrane‐controlled trial registry. The search terms specified the study population ‘cerebral palsy’ and the target intervention ‘surgical procedures’, or ‘surgery’, or ‘operative’. The reference lists of all included studies were searched to identify any potentially relevant studies. This search was supplemented by findings from two previous scoping reviews in this area published before 2016.^4^, ^21^

To be considered for inclusion, studies had to (1) report at least one outcome measure; (2) include children and young people with CP (aged from birth–20 years) and diagnosed with ambulatory CP, levels I to III of the GMFCS; (3) involve any lower limb orthopaedic surgery; and (4) be published in English. A detailed process of study selection and data extraction and synthesis has been published elsewhere.^3^ The breadth and depth of the content of included outcome measures were examined to support decision on suitability of the measures for the core outcome domains for lower limb orthopaedic surgical interventions that were identified earlier.^16^

### Step three: quality assessment of outcome measures

In accordance with the COSMIN guideline,^19^ judging the quality of an outcome measure requires evaluation of its psychometric properties. Nine measurement properties were defined and subdivided into three major groups; validity, reliability, and responsiveness.^19^, ^22^ The COSMIN guidance was used in this study, including a three‐stage appraisal process for each psychometric property: (1) methodological quality analysis; (2) level of evidence synthesis; and (3) best evidence synthesis.

#### Methodological quality analysis

The methodological quality of each study was rated with regard to the nine measurement properties.^23^ A four‐point scoring system was used: excellent, good, fair, and poor. This provided an overall score for the methodological quality of a study for each category of measurement property under examination. In line with the COSMIN guideline, the overall rating of each category was given as the lowest level response to any item in that category (psychometric property).^23^

#### Level of evidence synthesis

Level of evidence synthesis assessed whether the results for each measurement property were satisfactory.^24^ Three possible levels of evidence were used: positive (+), indeterminate (?), and negative (−). Where no information was available, a zero (0) rating was given. A description of these criteria is available in Appendix S1.

#### Best evidence synthesis

Best evidence synthesis rated the quality of the body of evidence on each property (Appendix S1). To examine the best evidence synthesis, the methodological quality rating for the measurement properties of each outcome measure was presented alongside the level of evidence in each study. This enabled the drawing of inferences on the relative robustness of evidence for each outcome measure.^19^

For this study, the COSMIN (http://database.cosmin.nl/) database was searched in July 2020 for studies that had investigated the psychometric properties of outcome measures in children with CP to determine the current state of the quality of the identified outcome measures in the field of CP lower limb orthopaedic surgery. The detailed process and methods are provided in Appendix S2.

### PubMed search

A further search was conducted in July 2020 through the sensitive PubMed search filter, as proposed by COSMIN, for measurement properties used^25^ for outcome measures that had no existing systematic review on their psychometric properties. Search key terms were (1) general names for outcome measures; (2) multidimensional health construct terms; (3) cerebral palsy; and (4) key terms relevant to psychometric performance. To ensure the transparency of the process, two researchers (HA and RK) independently conducted the search and assessed the quality of the studies. Any discrepancy was discussed, and consensus was reached through discussion between the researchers.

### Feasibility

The feasibility aspect of the outcome measure was also considered. This step required an evaluation of the interpretability, ease of administration, type and length of outcome measure, its completion time, costs, the equipment required to use it, type of administration, and other practical aspects. These feasibility criteria of each outcome measure were gathered from several resources such as the outcome measure's manual, primary development studies, or existing reviews.

### Data extraction

Data from the COSMIN database and the PubMed search were extracted using standardized, piloted data extraction in Microsoft Excel. For each included outcome measure, the following were extracted: name, abbreviation, the purpose of measure, number of items, age range, the responder (clinician, child and/or parent as proxy), response options, completion time, health domains assessed, methods for scoring, training requirement, COSMIN best evidence synthesis criteria for the nine psychometric properties, and the key reference.

### Step four: generic recommendations—consensus meeting

The evidence that was generated in steps 2 and 3 was used to inform a consensus process to decide whether or not the individual outcome measures were recommended for the assessment of the relevant core outcome domains. Ethical approval for the consensus meeting was gained from the relevant Research Ethics Committee (19/SC/0357).

Seven healthcare professionals with expertise in CP research and in assessing psychometric properties and usability of outcome measures were invited to take part. The meeting was held online in September 2021. The consensus meeting involved a combination of presentations of background information, and the roadmap was described to enable participants to understand fully how we had defined the outcome measures, followed by a presentation of the overview of the current state of the shortlisted outcome measures in terms of their quality and feasibility. A whole‐group discussion with the participants was undertaken, to reflect on the outcome measures. The goal was to identify the most optimal outcome measures and to consider their suitability in matching the outcome domains, on the basis of their psychometric properties and feasibility criteria. Given the relatively small expert panel size (*n* = 7), the group achieved consensus through collaborative discussion. Any areas of disagreement were resolved through iterative dialogue and clarification, allowing all panel members to contribute their expert perspectives until mutual agreement was reached on the final set of a core outcome measures. A consent was obtained from all the panellists to audio record the meeting and field notes to be taken to accurately link the profession associated with each discussion point made.

## RESULTS

### Identify existing outcome measures

In total, 354 eligible studies were identified which reported 43 different outcome measures. The output of the initial screening against pre‐specified criteria was a list of 20 outcome measures that were considered as potential core outcome measures. The list is shown in Appendix S3. Table 1 illustrates the mapping of the potential measures to the eight core outcomes (domains).

**TABLE 1:** Outcome measures aligned with the core outcome (domains) set.

Domain	Outcomes	Gait analysis			EVGS	PRS	OGS	FMS	GMFM	TUG	FAQ	PEDI	FIM	GMPM	MobQues	PODCI	PROMIS^a^	GOAL	CHQ	CP‐QoL	PedsQL
		GPS	GDI	GGI																	
Pain, discomfort, and fatigue	Pain															×	×	×	×	×	×
	Muscle fatigue																	×			
	Energy level after activities															×	×	×			×
Lower limb structure	Alignment and symmetry					×												×			
Motor function	Standing position								x									×			
	Fall and balance								x									×			×
Mobility: daily life activity	Basic life activities								×		×	×	×	×	×	×	×	×			
	Climbing stair								×		×		×		×	×	×	×			
Mobility: walking	Walking speed									×								×			
	Walking distance							×		×	×					×		×			
	Walking endurance											×			×	×		×			
	Walking pattern (quality)	×	×	×	×	×	×											×			
	Walking ability								×		x		×		×	×		×			
	Using orthosis, splint, and brace															×		×			
	Using assistive device							×				×				×		×			
Participation	Physical activity										×					×		×			
Independence	Independence												×					×			
Quality of life	Self‐esteem															x		×	×	×	
	Well‐being																		×	×	×

Abbreviations: CHQ, Child Health Questionnaire; CP‐QOL, Cerebral Palsy Quality of Life for Children; EVGS, Edinburgh Visual Gait Scale; Gillette FAQ, Gillette Functional Assessment Questionnaire; FIM, Functional Independence Measure; FMS, Functional Mobility Scale; GDI, Gait Deviation Index; GGI, Gillette Gait Index; GMFM‐66, Gross Motor Function measures; GMPM, Gross Motor Performance Measure; GOAL, Gait Outcomes Assessment List; GPS, Gait Profile Score; MobQues, Mobility Questionnaire; OGS, Observational Gait Scale; PEDI, Pediatric Evaluation of Disability Inventory; PedsQL, Paediatric Quality of Life Inventory; PODCI, Pediatric Outcomes Data Collection Instrument; PROMIS, Patient‐Reported Outcomes Measurement Information System; PRS, Physician Rating Scale; TUG, Time‐Up and Go.

^a^ PROMIS (pain interference, mobility) domains.

### Quality assessment of outcome measures

Nine systematic reviews evaluating the psychometric properties of the 17 outcome measures were identified in the COSMIN database. Twelve studies evaluating the psychometric properties of the three outcome measures (Gait Outcome Assessment List [GOAL], Pediatric Outcomes Data Collection Instrument, and Patient‐Reported Outcomes Measurement Information System [PROMIS]) were identified from the PubMed search and were appraised according to the COSMIN criteria (Appendix S4). The quality assessment, best evidence synthesis, and feasibility of the shortlisted 20 outcome measures are provided in Appendix S4.

### Consensus meeting recommendations

The consensus group recommended seven outcome measures to be used, where possible, in studies of lower limb orthopaedic surgery for ambulant children with CP. This core set of measures addressed comprehensively the previously determined set of eight core outcome domains. The recommended outcome measure(s) for each of the eight core outcome domains is discussed below and summarized in Figure S1 and Table 2. The psychometric properties and feasibility of these outcome measures are shown in Tables 3 and 4.^26^, ^27^, ^28^, ^29^, ^30^, ^31^, ^32^, ^33^, ^34^, ^35^, ^36^, ^37^, ^38^, ^39^, ^40^, ^41^, ^42^, ^43^, ^44^, ^45^, ^46^, ^47^, ^48^, ^49^, ^50^, ^51^, ^52^

**TABLE 2:** The recommended outcome measures for each core outcome domains.

Domain	Outcomes	Outcome measures
Pain, discomfort, and fatigue	Pain	GOAL, PROMIS (pain interference)
	Muscle fatigue	GOAL
	Energy level after activities	GOAL, PROMIS (fatigue)
Lower limb structure	Alignment and symmetry	GOAL
Motor function	Standing position	GOAL, GMFM‐66
	Fall and balance	GOAL
Mobility: daily life activity	Basic life activities	GOAL
	Climbing stair	GOAL
Mobility: walking	Walking speed	GOAL
	Walking distance	GOAL, Gillette FAQ
	Walking endurance	GOAL
	Walking pattern (quality)	3DGA, EVGS, GOAL
	Walking ability	Gillette FAQ, GOAL
	Using orthosis, splint, and brace	GOAL
	Using assistive device	GOAL
Participation	Physical activity	GOAL
Independence	Independence	GOAL
Quality of life	Self‐esteem	GOAL, CP‐QoL
	Well‐being	

Abbreviations: 3DGA, three‐dimensional gait analysis; CP‐QOL, Cerebral Palsy Quality of Life for Children; EVGS, Edinburgh Visual Gait Scale; Gillette FAQ, Gillette Functional Assessment Questionnaire; GMFM‐66, Gross Motor Function measures; GOAL, Gait Outcomes Assessment List; PROMIS, Patient‐Reported Outcomes Measurement Information System.

**TABLE 3:** Psychometric properties of the shortlisted outcome measures.

Outcome measure		Reliability			Validity					Responsiveness	
		Reliability	Measurement error	Internal consistency	Content validity	Structure validity	Hypothesis testing	Cross‐cultural validity	Criterion validity		References
3DGA	GGI	±	?	0	0	0	0	0	0	0	26
	GDI	?	?	0	0	0	+	0	0	?	26
	GPS	0	0	0	0	0	++	0	0	0	26
EVGS		?	0	0	0	0	+	0	?	?	26
GMFM‐66		+++	+	+++	+	+	+	0	+	+++	27, 28
Gillette FAQ		++	0	0	0	0	++	0	?	0	26, 29
CP‐QoL		+	0	?	0	?	+	0	0	0	30
GOAL		+++	0	+++	++	0	+++	++	++	+	10, 31, 32, 33, 34, 35, 36, 37
PROMIS		0	0	+	0	0	+	0	0	+	38, 39, 40

Abbreviations: 3DGA, three‐dimensional gait analysis; CP‐QoL, Cerebral Palsy Quality of Life for Children; EVGS, Edinburgh Visual Gait Scale; GDI, Gait Deviation Index; GGI, Gillette Gait Index; Gillette FAQ, Gillette Functional Assessment Questionnaire; GMFM‐66, Gross Motor Function Measure; GOAL, Gait Outcome Assessment List; GPS, Gait Profile Score; PROMIS, Patient‐Reported Outcomes Measurement Information System.

**TABLE 4:** Feasibility criteria of the shortlisted outcome measures.

Outcome measure		Administration format	Age	Administration time	Length of survey	Equipment needed	Assessor training	Cost	References
3DGA	GGI	Clinician	Not specified	Not specified	16 items	Gait lab, software	Yes	Lab: yes	41
	GDI	Clinician	Not specified	Not specified	9 items	Gait lab, software	Yes	Lab: yes	41
	GPS	Clinician	Not specified	Not specified	9 items	Gait lab, software	Yes	Lab: yes	41
EVGS		Clinician	Not specified	Not specified	17 items	Video recordings	Yes	Software: yes	42, 43, 44
GMFM‐66		Clinician	0–12 years	45–60 minutes	66 items	Computer, software	Yes	Manual: yes	45, 46
Gillette FAQ		Self‐ or proxy‐reported	Not specified	5 minutes	22 items	No	No	Free	8, 24
CP‐QoL		Self‐ or proxy‐reported	Self‐report: 9–12 years Proxy‐report: 4–12 years	15–25 minutes	Self‐report = 53 items Proxy‐report = 66 items	No	No	Free	47, 48
GOAL		Self‐ or proxy‐reported	9–18 years	12–19 minutes	48 items	Form	No	Free	10, 49
PROMIS		Self‐ or proxy‐reported	Self‐report: 8–17 years Proxy‐report: 5–17 years	Not specified	Not specified	Computer	No	Free	46, 50, 51, 52

Abbreviations: 3DGA, three‐dimensional gait analysis; CP‐QoL, Cerebral Palsy Quality of Life for Children; EVGS, Edinburgh Visual Gait Scale; GDI, Gait Deviation Index; GGI, Gillette Gait Index; Gillette FAQ, Gillette Functional Assessment Questionnaire; GMFM‐66, Gross Motor Function Measure; GOAL, Gait Outcome Assessment List; GPS, Gait Profile Score; PROMIS, Patient‐Reported Outcomes Measurement Information System.

### Domain 1: pain, discomfort, and fatigue

The consensus group felt that the pain location and pain interference with the child's daily life were essential aspects of pain to consider in the evaluation of outcomes. Two measures reached consensus: the GOAL^9^ and PROMIS (computer adaptive tests for pain interference).^50^ The GOAL questionnaire was favoured over the PROMIS because the GOAL is a condition‐specific measure designed to evaluate the effectiveness of surgical interventions in children with CP. Although condition‐specific measures may be more sensitive to change, the PROMIS, which is a generic measure, was recommended as the preferred measure for comparison of the CP population with other paediatric orthopaedic conditions.

On the basis of the consensus panel's discussion about fatigue outcomes, addressing fatigue is a significant concern for children with CP. While the GOAL measure was recommended as the best among the available measures, the panel recommended a desire for a more comprehensive assessment that separates fatigue from pain. The panel emphasized the need for additional research and potentially more focused outcome measures. Similar to the pain measures, the panel believed incorporating a generic measure for fatigue, such as the PROMIS (fatigue), could enhance comparability with other conditions, providing a broader context for understanding disability and functional levels in children with CP.

### Domain 2: lower limb structure

The ‘lower limb structure’ domain describes the appropriate alignment and symmetry of the lower limbs, both functionally and cosmetically. It is important to note that improvement of lower limb structure is the primary purpose of the surgical interventions in this field. A consensus was reached on using the GOAL questionnaire on the basis that this reflects importance of limb structure correction for the children and their parents.

### Domain 3: motor function

Consensus was on using the Gross Motor Function Measure (GMFM‐66)^45^ and the GOAL questionnaire as clinician‐driven and patient‐reported outcome measures respectively. Panel members reflected on the time taken to complete the assessment with the GMFM‐66 and the training required to complete it. However, the panel felt that the reliability, validity, and depth of the GMFM‐66 content to capture positioning outcome in the motor function domain outweighed these disadvantages. The GOAL was recommended to complement the clinician‐rated measure (i.e. GMFM‐66).

### Domain 4: daily life activities

The consensus group felt that it was important to understand the impact of surgical interventions on the child's daily life activities. The opinions of the children and their parents or carers were considered important in this respect. Therefore, the GOAL questionnaire reached consensus as the most appropriate patient‐reported outcome measure that was appropriate in assessing aspects of daily life activities. The panel believed there was potential for recommending the Mobility Questionnaire (MobQues47) and Functional Independence Measure tools in the future, depending on the results of further validation studies.

### Domain 5: gait‐related outcomes

The three‐dimensional gait analysis (3DGA),^41^ Edinburgh Visual Gait Scale (EVGS),^42^ the Gillette Functional Assessment Questionnaire (Gillette FAQ),^29^ and the GOAL questionnaire reached consensus as clinician‐driven and patient‐reported outcome measures appropriate for measuring mobility. These cover all aspects of gait‐related outcomes (e.g. speed, distance, and pattern).

The 3DGA is the criterion standard in surgical decision‐making for children with CP because it provides comprehensive information on joint movement and loading. However, it requires particular expertise in gait data interpretation and extensive resources for a gait laboratory. The panel agreed that the EVGS^42^ was a useful clinician‐driven alternative to 3DGA in settings where these resources are limited or not available. The panel did not discuss whether gait tests should be undertaken at preferred or fastest walking speed. The Gillette FAQ and the GOAL are both patient‐reported outcome measures and both capture important aspects of mobility outcomes. The panel members recommended the GOAL measure over the Gillette FAQ, as the former provided additional items. These assessed the level of assistance that was required for different activities and covered orthotic aspects. Similarly, the panel acknowledged that the GMFM‐66 and Pediatric Evaluation of Disability Inventory demonstrate a good content validation study. However, after discussion, these measures were not recommended to measure gait because of concern about their feasibility (administration time) and that they may lead to respondent burden.

### Domain 6: physical activity

Physical activity describes a child's ability to engage in typical age‐specific activities. The panel recommended the GOAL patient‐reported outcome measure as the best measurement of ‘physical activity’. The GOAL includes eight questions that specifically relate to physical activities.

### Domain 7: independence

No previous study has specifically explored the impact of lower limb orthopaedic surgery on the child's level of independence. The GOAL and Pediatric Outcomes Data Collection Instrument cover aspects of the level of independence in mobility, including self‐care, mobility indoors (home), walking, using stairs, and physical activity. The panel recommended using the GOAL questionnaire as a measurement of ‘independence’, because it includes 19 questions that relate to daily life activities and gait‐related outcomes relevant to independence. However, assessors should consider the level of assistance required when interpreting the results, as it provides important context beyond the total or domain scores. Additionally, the panel believed the GOAL had advantages above the other measures, as it measures the level of happiness and satisfaction in using assistive devices.

### Domain 8: quality of life

The panel discussed the difficulties in measuring QoL owing to its multidimensionality and felt that self‐esteem and body image contributed to it. The group discussion focused on the variability of domains in QoL outcome measures and agreed that no single outcome measure could cover all aspects of QoL. Members emphasized that different outcome measures should be suggested and used in different settings, depending on the research question under study. Since the panel felt unable to suggest a single preferred outcome measure specific to QoL, two outcome measures were recommended: the GOAL and the Cerebral Palsy Quality of Life for Children (CP‐QoL) questionnaires.^47^ The CP‐QoL list is a comprehensive tool that records QoL in relation to general health, participation, communication, pain, family and friends, school, use of special equipment, and access to services.

### Updated literature search

Following the consensus group meeting (September 2021) and recommendations, we conducted an updated search of the literature using the original search terms to identify any recent relevant studies on the psychometric studies of the identified outcome measures. The search was updated for literature published in the COSMIN and PubMed databases from August 2020 to July 2024. Five new studies were identified (Appendix S5): one evaluated the psychometric properties of the CP‐QoL questionnaire and four evaluated the psychometric properties of the GOAL questionnaire.

The included studies in the GOAL questionnaire demonstrated strong psychometric properties (reliability and validity) across multiple quality domains, with the exception of concurrent validity, where further research is warranted. Overall, the findings from this updated search did not alter the recommendations made in 2021; more specifically, the studies included in the updated search tended to favour the GOAL questionnaire.

## DISCUSSION

This study presents recommendations for a core set of outcome measures to be used in research related to lower limb orthopaedic surgery for ambulant children with CP. We have previously developed a set of core outcome domains to be used in research in this field of clinical practice.^16^ The process of the present study involved a review to identify all available measures to cover the previously defined domains^3^ and a subsequent assessment of their psychometric properties and feasibility. This was followed by a consensus meeting on the most appropriate outcome measures. The outcome measures recommended by the consensus group were a combination of clinician‐driven measures: the 3DGA, EVGS, and GMFM‐66; and of patient‐reported measures: the GOAL, Gillette FAQ, PROMIS, and CP‐QoL.

Schiariti et al.,^46^ Wright et al.,^53^ and Fong et al.^11^ suggested that using a combination of clinician‐ and patient‐reported outcomes is appropriate and important. Some measures, when used alone, may fail to capture the real impact of surgery on the child owing to the variety of the parameters that may influence the outcomes of interest. One example is the measurement of gait deviations through the use of 3DGA, which is a gait‐specific outcome measure and important in surgical decision‐making.^54^, ^55^ Studies have shown that the postoperative improvements of gait deviations at 1 year from treatment, as measured by 3DGA, are correlated moderately or poorly with patient satisfaction and perceived QoL.^56^ When future clinical trials are designed, relevant outcome measures should assess the multiple aspects of the surgical outcome and reflect performance in a real‐life context. Patient‐reported outcome measures should occupy a central place in the selection of outcome measures, alongside clinician‐driven measures.

The preferred outcome measure in the consensus panel was the GOAL, as it reflected well outcomes across the International Classification of Functioning, Disability and Health (ICF) for Children and Youth framework. This had been recommended in previous reviews in this field. For example, the review of outcome measures in this field by Wilson et al.^4^ stated, ‘We suggest that in order to understand the full impact of lower limb orthopaedic surgery, a suite of outcome measures across the ICF may be needed, including the domain of activity and participation that reflect outcomes relevant to the patient, family, and surgeon’. Watkins et al.^2^ stated that ‘no single instrument can be used to measure all of the components of the ICF’ when they reviewed functional and health‐related QoL measures for children with CP, and recommended use of multiple measures to cover aspects of the ICF.

Some reliability and validity of the GOAL have been rated as ‘limited’ or ‘unknown’; however, this is to be expected because it was a newly developed measure at the time of the review, and there was a lack of primary evaluation of its psychometric properties. However, further empirical studies have been developed and published since our review to assess the quality and strength of the GOAL questionnaire in the context of reliability testing, validation, cultural adaptation in different languages (e.g. German), and responsiveness to change after selective dorsal rhizotomy.^31^, ^57^, ^58^ The findings of these studies added value to the knowledge of the psychometrics properties of the GOAL questionnaire.

The inclusion of the GOAL measure^10^ was strongly recommended for inclusion in the core set of outcome measures. First, the use of the measure enables the research/practice agenda to move beyond a focus on symptom management/disability and to consider the role of surgery in other health domains, such as gait‐related outcomes, physical activity, and the QoL of children with CP. Second, it reflects the awareness of researchers and clinicians of the outcomes that are relevant to the patient and family, that is, towards activity and participation.

The goal of this study was to identify the best available outcome measures suitable for the previously agreed core outcome domains set. The selection of the outcome measures was based on the best evidence available; however, some of the recommended outcome measures require further work to better understand their psychometric properties. Additionally, we note that our proposed outcome measures include only indirect assessment of fall risks. A more accurate and direct way to assess risk of falling would include measuring fall risk and its impact on the child's activities. This should be considered in future research focusing on fall risks.

Apart from the 3DGA, the core outcome measures that were recommended are commonly used assessments that can be completed relatively quickly, require no specialized equipment or training (with the exception of the GMFM‐66), and have minimal cost implications. It should therefore be feasible to implement these outcome measures in future studies on lower limb orthopaedic surgery. It is hoped that the simplicity and flexibility of the recommended measures will help promote uptake of this core set. Although this core set was designed for research, its simplicity would make it suitable for use in clinical practice, in monitoring the progress and measuring change throughout surgical treatment and recovery.

With regards to gait analysis, kinematic and kinetics parameters and their derivatives in the form of Gait Profile Score and Gait Deviation Index values are the most commonly used gait measures; more research is needed to determine the most relevant gait parameters derived from the 3DGA, and the clinical examination as outcome measures. It should also be borne in mind that the validity and reliability of 3DGA measurements depends on the quality control practices of the relevant labs producing them. Critical appraisal and scientific rigor in evaluating these parameters is, therefore, required. However, in the absence of 3DGA, observational gait assessment using the EVGS can serve as a valuable alternative measure for assessing gait‐related outcomes after surgical interventions. This aligns with the recommendation by Fong et al.,^11^ who considered the use of EVGS as one of the core outcome measures in gait corrective orthopaedic surgery for CP.

The recommended set of core outcome measures covers comprehensively and reliably the previously defined core outcome domains.^16^ It is advisable to administer these measures at baseline and at different time points during follow‐up depending on the type of treatment delivered, as recommended by COMET and COSMIN initiatives.^19^ To ensure consistency and less heterogeneity in the evidence base, researchers should be encouraged to use this core set of measures in future research to assess and report the impact of surgery on the domains of interest. In addition, by using the same measures, it will become easier to interpret evidence across different studies, trials, and meta‐analyses, leading to evidence‐based recommendations and clinical guidelines. This approach should have positive implications for both value‐driven care and quality improvement perspectives.

It is not intended that these recommended measures should be the only ones for use in future studies, but that they may be supplemented with other measures according to the purpose of the study, for example whether the study is for exploratory purposes or to evaluate condition‐specific issues. In light of the evolving evidence base, the publication of new outcome measures and the continued validation/update of existing outcome measures (e.g. short form), the proposed core set of outcome measures must be reviewed regularly. During such review, the results of recent studies may provide stronger evidence to support the inclusion of these proposed measures or additional different ones.

The high implementation cost and global application of the proposed core set, particularly in low‐ and middle‐income countries, may affect its potential for adoption.^59^, ^60^ First, the outcome measures included in our core set would require translation and validation in different languages, if not available. Second, although, some outcome measures may be applicable both to low‐ and middle‐income countries and to high‐income countries, there may be significant differences in healthcare system capabilities and resource availability. We hope that the process of applying this core outcome set to low‐ and middle‐income countries could support the development of healthcare strategies to improve outcomes in those countries.

A strength of our study is that it captures and aids the implementation of the previously developed core outcome domains set of lower limb orthopaedic surgery for ambulant children with CP.^16^ Additionally, it provides a set of core outcome measures for enhancing the quality and consistency of reporting outcome measures in future studies and clinical trials. The consensus decisions were made on the basis of best evidence on lower limb surgery for ambulant children with CP literature that included recently published systematic reviews on the psychometric properties and feasibility criteria. Moreover, the consensus methodology was informed by existing recommendations and guidelines such as those produced by the COMET and COSMIN initiatives.

There were several limitations to our study. First, there was the specificity of the outcome measures identified during the initial scoping review, which focused primarily on studies related to lower limb orthopaedic surgery. As a result, some relevant outcome measures commonly used in broader clinical practice may not have been captured. This may affect the overall comprehensiveness of the proposed core outcome set, as it may not fully reflect the wide range of measures used to assess the core domains across different settings and populations. Second, the consensus meeting panel was small, and its members were predominantly from the UK and other English‐speaking countries. This may have affected the consensus process and findings. Third, patients were not involved in the consensus procedure, as this is not part of the COMET/COSMIN recommended methodology. Patients and representatives (e.g. parents) could have provided useful comments on the usability and comprehension of the outcome measures. However, it should be stressed that it is unclear how patients could contribute to the selection of core outcome measures and consider aspects such as psychometric properties, as methodological research in this field is lacking. Fourth, all the core measures are in English formats, and some have not been validated in other languages, which may limit the applicability of the core outcome measurement set, particularly the patient‐reported outcome measures. In future, research is necessary with respect to the cultural adaptation and validation in a range of different languages to support the use of the core outcome measurement set in countries where English is not the principal language.

## CONCLUSION

It is recommended that a combination of patient‐reported and clinician‐driven outcome measures should be used when reporting outcomes of lower limb orthopaedic surgery on ambulant children with CP. Practical aspects (e.g. costs) and availability of these measures in a particular language or country should be considered. The use of a core set of outcome measures has the potential to enhance greatly the validity, comparability, and clinical applicability of clinical trials of lower limb orthopaedic surgical interventions.

Feedback on the relevance and acceptability of the recommended outcome measures in real‐life research will be essential to ensure that meaningful outcomes are captured in future studies.

## CONFLICT OF INTEREST STATEMENT

The authors have stated that they had no interests that might be perceived as posing a conflict or bias.

## Supporting information


**Appendix S1:** Criteria for good measurement properties.

**Appendix S2:** COSMIN database.

**Appendix S3:** Criteria for initial reduction of the number of outcome measurement instruments.

**Appendix S4:** Characteristics of the included studies and ratings of methodological quality and quality of evidence.

**Appendix S5:** Updated search (August 2020–July 2024).

**Figure S1:** Flowchart of the development of the core outcome measures set.

## Data Availability

The data that supports the findings of this study are available in the supplementary material of this article.
