# Supplementary material for: Development of a set of core outcome measures for ambulant children with cerebral palsy after lower limb orthopaedic surgery
Source: Dev Med Child Neurol. 2025 Dec 29;68(8):1127–38. doi: 10.1111/dmcn.70133 (PMC13340623; doi:10.1111/dmcn.70133)
Supplement: Supplementary file 1 — Appendix S1: Criteria for good measurement properties. [file DMCN-68-1127-s006.pdf]

Table 1 Criteria for good measurement properties

Term		Rating	Definition
**Domain**	**Measurement property**		
**Validity**	Content validity (Including face validity)	+	All items refer to relevant aspects of the construct to be measured AND are relevant for the target population AND are relevant for the context of use AND together comprehensively reflect the construct to be measured
		?	Not all information for '+' reported
		–	Criteria for '+' not met
	Structure validity	+	Factors should explain at least 50% of the variance
		?	Explained variance not mentioned
		–	Criteria for '+' not met
	Construct validity hypothesis testing	+	At least 75% of the results are in accordance with the hypotheses
		?	No correlations with instrument(s) measuring related construct(s) AND no differences between relevant groups reported
		–	Criteria for '+' not met
	Cross-cultural validity	+	No important differences found between language versions in multiple group factor analysis or DIF analysis
		?	Multiple group factor analysis AND DIF analysis not performed
		–	One or more criteria for '+' not met
	Criterion validity	+	Convincing arguments that gold standard is "gold" AND correlation with gold standard ≥ 0.70
		?	Not all information for '+' reported
		–	Criteria for '+' not met
**Reliability**	Reliability	+	ICC or weighted Kappa ≥ 0.70 or Pearson's r≥ 0.80
		?	ICC or weighted Kappa not reported
		–	Criteria for '+' not met
	Measurement error	+	SDC or LoA < MIC
		?	MIC not defined
		–	Criteria for '+' not met
	Internal consistency	+	Cronbach's alpha(s) ≥ 0.70 and ≤ 0.95
		?	Cronbach's alpha not determined or dimensionally unknown
		–	Criteria for '+' not met
**Responsiveness**	Responsiveness	+	At least 75% of the results are in accordance with the hypotheses
		?	No correlations with changes in instrument(s) measuring related construct(s) AND no differences between changes in relevant groups reported
		–	Criteria for '+' not met

ICC = intraclass correlation coefficient, LoA = limits of agreement, MIC = minimal important change, SDC = smallest detectable change
* "+" = positive rating, "?" = indeterminate rating,"– "= negative rating

Table 2 Quality of evidence

Quality Rating	Criteria
Strong (+++)	Consistent findings in multiple studies of at least good quality OR one study of excellent quality AND a total sample size of ≥100 patients
Moderate (++)	Consistent findings in multiple studies of at least good quality OR consistent findings in multiple studies of at least fair quality OR one study of good quality AND a total sample size of ≥50 patients
Limited (+ or -)	Limited findings in multiple studies of at least fair quality OR one study of fair quality AND a total sample size of ≥30 patients
Conflicting (±)	Conflicting findings
Poor (?)	Only studies of poor quality OR a total sample size of <30 patients
Unknown (0)	No studies
