# Supplementary material for: Development of a set of core outcome measures for ambulant children with cerebral palsy after lower limb orthopaedic surgery
Source: Dev Med Child Neurol. 2025 Dec 29;68(8):1127–38. doi: 10.1111/dmcn.70133 (PMC13340623; doi:10.1111/dmcn.70133)
Supplement: Supplementary file 2 — Appendix S2: COSMIN database. [file DMCN-68-1127-s001.pdf]

COSMIN Database

The COSMIN database search initially identified 58 reviews. The titles and abstracts of these reviews were extracted and screened. Of these, 49 were excluded: five used different appraisal tools (e.g., CanChild rating form), 12 did not provide data regarding relevant outcome measures, two were studies of primary psychometric properties, 20 were out of the current review scope, six were structured/ scoping reviews of literature, one was based on outcome measures in the adult CP population, and three reviews did not specify the health conditions that had been involved in the studies (e.g., children with neurological disorders). An example of the excluded reviews can be found in Table 3. This process left nine reviews for inclusion. Table 4 provides a summary of the characteristics of the included reviews.

The identified reviews were published between 2008 and 2020. The majority of the reviews were related to children with CP (n= 5). The reviews had diverse focus: three targeted gait- and walking-related outcomes, two aimed to assess quality of life outcome measures, two reviews focused on outcome measures of motor function, one focused on balance and one focused on daily life activities.

Table 1 COSMIN Database: Excluded reviews

Review	Ref
Appraised by different tools	
A systematic review of the psychometric properties of Quality-of-Life measures for school aged children with cerebral palsy	1
Did not evaluate relevant outcome measure	
Measuring quality of movement in cerebral palsy: a review of instruments	2
Primary psychometric properties study	
A systematic review of utility values in children with cerebral palsy	3
Out of the review scope	
Tactile assessment in children with cerebral palsy: a clinometric review	4
Structure/ scoping review	
Child or family assessed measures of activity performance and participation for children with cerebral palsy: a structured review	5
Outcome measures for Adults with CP	
How to measure pain in neurological conditions? A systematic review of psychometric properties and clinical utility of measurement tools	6
Did not specify health condition	
Fatigue in child chronic health conditions: a systematic review of assessment instruments	7

Table 2 Reviews identified from COSMIN database

Author/ year	Title	Condition	Review Focus	Outcome measures	Ref
Pavao et al (2017)	Clinical tools designed to assess motor abilities in children with cerebral palsy	CP	Motor abilities	GMPM, PEDI, FIM	8
Saether et al (2013)	Clinical tools to assess balance in children and adults with cerebral palsy: a systematic review	CP	Balance	TUG	9
Ferre-Fernándes et al (2020)	Measures of Motor and Functional Skills for Children with Cerebral Palsy: A Systematic Review	CP	Motor function and skills	GMPM, FIM	10
Himuro et al (2017)	Easy-to-use clinical measures of walking ability in children and adolescents with cerebral palsy: a systematic review	CP	Walking ability	TUG, FMS, Gillette FAQ	11
Zanudin et al (2017)	Psychometric properties of measures of gait quality and walking performance in young people with Cerebral Palsy: A systematic review	CP	Gait quality and walking performance	3DGA (GDI, GPS), OGS, EVGS, PRS, TUG, FMS, Gillette FAQ	12
Debuse et al (2011)	Outcome Measures of Activity for Children with Cerebral Palsy: A Systematic Review	CP	Activity	GMFM, PEDI	13
Ammann-Reiffer et al (2014)	Measurement properties of gait-related outcomes in youth with neuromuscular diagnoses: a systematic review	Neuromuscular diagnoses	Gait function	MobQues47	14
Janssens et al (2015)	A Systematic Review of Generic Multidimensional Patient-Reported Outcome Measures for Children, Part II: Evaluation of Psychometric Performance of English-Language Versions in a General Population	Children and young adult (all health condition)	Quality of Life	CHQ, PedsQL	15
Coombes et al (2016)	Health-related quality-of-life outcome measures in paediatric palliative care: A systematic review of psychometric properties and feasibility of use	Paediatrics	Quality of Life	CP-QoL	16

References

1. Carlon S, Shields N, Yong K, Gilmore R, Sakzewski L, Boyd R. A systematic review of the psychometric properties of Quality of Life measures for school aged children with cerebral palsy. *BMC Pediatr*. Nov 9 2010;10(81):1-11. doi:10.1186/1471-2431-10-81
2. Boyce WF, Gowland C, Rosenbaum PL, et al. Measuring quality of movement in cerebral palsy: a review of instruments. *Phys Ther*. Nov 1991;71(11):813-9. doi:10.1093/ptj/71.11.813
3. Tonmukayakul U, Le LK, Mudiyanselage SB, et al. A systematic review of utility values in children with cerebral palsy. *Qual Life Res*. Jan 2019;28(1):1-12. doi:10.1007/s11136-018-1955-8
4. Auld ML, Boyd RN, Moseley GL, Johnston LM. Tactile assessment in children with cerebral palsy: a clinimetric review. *Phys Occup Ther Pediatr*. Nov 2011;31(4):413-39. doi:10.3109/01942638.2011.572150
5. Morris C, Kurinczuk JJ, Fitzpatrick R. Child or family assessed measures of activity performance and participation for children with cerebral palsy: a structured review. *Child Care Health Dev*. Jul 2005;31(4):397-407. doi:10.1111/j.1365-2214.2005.00519.x
6. Tyson SF, Brown P. How to measure pain in neurological conditions? A systematic review of psychometric properties and clinical utility of measurement tools. *Clin Rehabil*. Jul 2014;28(7):669-86. doi:10.1177/0269215513514231
7. Crichton A, Knight S, Oakley E, Babl FE, Anderson V. Fatigue in child chronic health conditions: a systematic review of assessment instruments. *Pediatrics*. Apr 2015;135(4):e1015-31. doi:10.1542/peds.2014-2440
8. Pavão SL, Silva FPS, Dusing SC, Rocha NACF. Saleh Clinical tools designed to assess motor abilities in children with cerebral palsy. *Dev Neurorehabil*. 2017/04/03 2017;20(3):149-159. doi:10.3109/17518423.2016.1150359
9. Saether R, Helbostad JL, Riphagen, II, Vik T. Clinical tools to assess balance in children and adults with cerebral palsy: a systematic review. *Dev Med Child Neurol*. Nov 2013;55(11):988-99. doi:10.1111/dmcn.12162
10. Ferre-Fernández M, Murcia-González MA, Barnuevo Espinosa MD, Ríos-Díaz J. Measures of Motor and Functional Skills for Children With Cerebral Palsy: A Systematic Review. *Pediatr Phys Ther*. 2020;32(1):12-25.
11. Himuro N, Abe H, Nishibu H, Seino T, Mori M. Easy-to-use clinical measures of walking ability in children and adolescents with cerebral palsy: a systematic review. *Disabil Rehabil*. May 2017;39(10):957-968. doi:doi:10.1080/09638288.2016.1175036
12. Zanudin A, Mercer TH, Jagadamma KC, van der Linden ML. Psychometric properties of measures of gait quality and walking performance in young people with Cerebral Palsy: A systematic review. *Gait Posture*. Oct 2017;58:30-40. doi:doi:10.1016/j.gaitpost.2017.07.005
13. Debuse D, Brace H. Outcome measures of activity for children with cerebral palsy: a systematic review. *Pediatr Phys Ther*. Fall 2011;23(3):221-31. doi:10.1097/PEP.0b013e318227bbc6
14. Ammann-Reiffer C, Bastiaenen CHG, de Bie RA, van Hedel HJA. Measurement Properties of Gait-Related Outcomes in Youth With Neuromuscular Diagnoses: A Systematic Review. *Phys Ther*. 2014;94(8):1067-1082. doi:10.2522/ptj.20130299
15. Janssens A, Rogers M, Thompson Coon J, et al. A systematic review of generic multidimensional patient-reported outcome measures for children, part II: evaluation of psychometric performance of English-language versions in a general population. *Value Health*. Mar 2015;18(2):334-45. doi:10.1016/j.jval.2015.01.004
16. Coombes LH, Wiseman T, Lucas G, Sangha A, Murtagh FEM. Health-related quality-of-life outcome measures in paediatric palliative care: A systematic review of psychometric properties and feasibility of use. *Palliat Med*. 2016/12/01 2016;30(10):935-949. doi:10.1177/0269216316649155
