# Supplementary material for: Development of a set of core outcome measures for ambulant children with cerebral palsy after lower limb orthopaedic surgery
Source: Dev Med Child Neurol. 2025 Dec 29;68(8):1127–38. doi: 10.1111/dmcn.70133 (PMC13340623; doi:10.1111/dmcn.70133)
Supplement: Supplementary file 3 — Appendix S3: Criteria for initial reduction of the number of outcome measurement instruments. [file DMCN-68-1127-s005.pdf]

Criteria for initial reduction of the number of outcome measurement instruments

**Measures were excluded if:**
1. The purpose of the measurement instrument was to screen/set a goal for an intervention, rather than to measure outcomes
2. The measurement instrument was published more than 30 years ago (i.e., prior to 1990) without subsequent revision and/or was not in current use
3. Part of other measures

Outcome measure	Wilson (2014)	Wilson (2015)	Almoajil (2020)	Inclusion	Exclusion/ justification
**Body Function/ Structure**					
Clinical examination	x	x	x	In	Part of Gait Analysis
Gait analysis	x	x	x	In	
Gait velocity	x	x	x		
Movement Analysis Profile			x		
Edinburgh Visual Gait Score			x	In	
Radiology	x	x	x	In	
Type of walking device	x				Subjective
Surface electromyography	x	x		In	
Presence of pain	x	x			Subjective
Foot pressure data	x	x	x		
Physiological Cost Index	x				The measurement instrument was published more than thirty years ago without subsequent revision and/or was not in current use
Energy cost of walking/ oxygen consumption	x				
Biomechanical Model	x				
Normalcy Index	x				
Hip Flexor Index	x				
Observation gait	x				
Vertical plantar pressure	x				
Selective Control	x				
Assessment of the Lower Extremity	x				
Total mechanical work		x			
Physician Rating Scale	x			In	
Timed Up and Go		x	x	In	
**Activity and participation**					
Gross Motor Function Measure	x	x	x	In	
Gillette Functional Assessment Questionnaire	x	x	x	In	
Functional Mobility Scale	x	x	x	In	
Functional Independence Measure for Children	x	x		In	
Positional Activity Logger	x			In	
Pediatric Evaluation of Disability Inventory	x	x	x	In	
Mobility Questionnaire 47			x	In	
Modified Goal Attainment scale	x				The purpose of the measurement instrument was to set a goal only for general CP population
Gross Motor Performance Measure	x			In	
**Quality of life**					
Child Health Questionnaire	x			In	

Pediatric Quality of Life Inventory	x			In	
Cerebral Palsy Quality of Life for Children		x		In	
**Multi-dimensional measures**					
Pediatric Outcomes Data Collection Instrument	x	x	x	In	
Computerised adaptive test			x		Part of PROMIS
PROMIS			x	In	
**Further outcome measures**					
GOAL				In	
DISABKIDS- CP				In	
