# Supplementary material for: Development of a set of core outcome measures for ambulant children with cerebral palsy after lower limb orthopaedic surgery
Source: Dev Med Child Neurol. 2025 Dec 29;68(8):1127–38. doi: 10.1111/dmcn.70133 (PMC13340623; doi:10.1111/dmcn.70133)
Supplement: Supplementary file 4 — Appendix S4: Characteristics of the included studies and ratings of methodological quality and quality of evidence. [file DMCN-68-1127-s002.pdf]

Table 1 Characteristics of the included studies and ratings of methodological quality and quality of evidence

Outcome measure	Study	n, age (y)	Psychometric properties	Results	(COSMIN) rating	Quality of Evidence	Ref
GOAL	Thomason et al (2018)	105, 6-20	Hypothesis testing: discrimination validity	(Mean [SD]) GMFCS level I, 72.5 [12.7]; GMFCS level II, 61.4 [13.0]; GMFCS level III, 38.8 [10.6]; [F2,97=42.4, p<0.001])	Excellent	+	1
			Construct validity	FMS (5m and 50m r=0.59; 500m r=0.66) FAQ walking (r=0.77) and activities list (r=0.75, p<0.01). GPS (r=0.59) and gait appearance domain and GPS (r=0.52, p<0.01)	Excellent	+	
	Thomason et al (2020)	43, 6-23	Responsiveness	Surgical group: mean (SD) 61.7 (18.1) to 63.8 (16.1) Non-surgical group: mean (SD) 65.5 (15.6) to 64.6 (16.4) Correlation between change in total GOAL and GPS for all children was −0.42. The strongest correlation was −0.47 between the gait appearance domain and GPS	Good	+	2
	Karpati et al (2015)	51, 7-16	Content validity	Children's priorities correlated with their parents'; however, parents rated all items as more important goals than their children	Poor	+	3
	Boyer et al (2021)	310, 3-25	Hypothesis testing: convergent validity	95% CI GMFCS I-IV= 0.73-0.84	Excellent	+	4
			Construct validity	95% CI GMFCS I-IV= 0.24-0.49	Excellent	+	
PODCI	Damino et al (2005)	35, 4-16	Responsiveness	Surgical group: the GMFM Total score was responsive to change after both MT surgery, p < 0.05 for pre/postsurgery. Significant correlations found between PODCI Global Function Scale and Subscales and GMFM Scores were mostly in the moderate to high range (0.64-0.94), with the highest value seen between the GMFM Total Score and the Transfers and Basic Mobility Subscale.	Excellent	+	5
	Deltroy et al (1998)	470, 2-8	Internal consistency	Cronbach's alpha 0.76–0.97	Excellent	+	6
			Interrater reliability	0.45–0.87, highest for physical domain (parent vs child)	Good	-	
			Test-retest reliability	0.71–0.9	Good	+	
	McCarthy et al (2002)	115, 3-10	Hypothesis testing: convergent validity	Spearman's partial correlation coefficients 0.60–081 (CHQ), 0.56–0.94 (GMFM), 0.50–0.81 (PEDI)	Excellent	+	7
			Internal consistency	Cronbach's alpha 0.76–0.97	Excellent	+	
			Hypothesis testing: discrimination validity	Detect change according to severity: The transfer and mobility subscale (RV, 62%)	Excellent	+	
	Pencharz et al (2001)	166, 5-17	Construct validity: convergent validity	Spearman's partial correlation coefficients CHQ: r ≥ 0.68 (pain subscale) CHQ: 0.21 ≤ r ≤ 0.56 (global function) ASK: r ≥ 0.78	Good	+	8
			Hypothesis testing:	Detect change according to topographic: F test (74.8) 0.0001 ≤ p ≤ 0.005	Excellent	+	

			discriminative validity				
	Vitale et al (2005)	180, 5-18	Hypothesis testing: convergent validity	Sensitive to differences in the diplegic and hemiplegic groups	Excellent	+	9
			Criterion validity	% Agreement (physical score): 33% - 41% (except diplegia)<br>% Agreement (psychological score): 8% - 35%	Poor	?	
PROMIS	Kratz et al (2013)	82, 8-19	Internal consistency	Cronbach's alpha 0.75	Excellent	+	10
			Construct validity: convergent validity	Scores of PROMIS (short form) and PROMIS mobility CAT, respectively<br>PedsQL: 0.58-0.60<br>PODCI (mobility): 0.52-0.39<br>PODCI (sport): 0.60-0.49<br>Gillette FAQ: 0.48-0.41<br>GMFM (stand): -39-0.21)<br>GMFM (walk): 0.39-0.19<br>TUG: -30 - -0.16	Excellent	+	
			Hypothesis testing	PROMIS (Short Form) and all legacy measures were able to distinguish between GMFCS categories<br>F (2,81) =9.55 p <0.001<br><br>PROMIS (CAT) scores did not show significant mean differences between the groups<br>F (2,78) = 2.90 p=0.06	Excellent	-	
	Coster et al (2016)	303, 8-17	Structure validity	Fatigue, M2=1050.25, (RMSEA)=0.04; Pain Interference, M2=806.99, RMSEA=0.05. Mobility, M2=638.6, RMSEA=0.04.	Good	-	11
	Mulcahey et al (2016)	Baseline: 113, 12.7 (3.0)<br>6-month: 98, 12.7 (3.1)<br>12-month: 86, 12.4 (3.0)	Responsiveness	The PROMIS CAT and SF did not detect change at either the 6- or 12- months. ES and SRM for all PROMIS Pediatric Measures were non-significant.	Excellent	+	12

GOAL: Gait Outcome Assessment List, PODCI: Pediatric Outcomes Data Collection Instrument PROMIS: Patient-Reported Outcomes Measurement Information System.

Table 2 Psychometric properties of the shortlisted outcome measures.

Outcome Measure		Reliability			Validity					Responsiveness	References
		Reliability	Measurement error	Internal Consistency	Content validity	Structure validity	Hypothesis testing	Cross-cultural validity	Criterion validity		
Gait Analysis	GGI	±	?	0	0	0	0	0	0	0	13
Gait Analysis	GDI	?	?	0	0	0	+	0	0	?	13
Gait Analysis	GPS	0	0	0	0	0	++	0	0	0	13
EVGS		?	0	0	0	0	+	0	?	?	13
OGS		?	0	0	0	0	0	0	-	0	13
PRS		±	0	0	0	0	0	0	0	0	13
GMFM		+++	0	0	++	0	+	0	0	+++	14
FMS		++	0	0	0	0	?	0	?	+	13, 15
Gillette FAQ		++	0	0	0	0	++	0	?	0	13, 15
PEDI		+++	0	+++	++	+	+++	+	0	+++	14, 16
GMPM		++	0	0	0	0	+++	0	0	?	16, 17
FIM		+	0	+++	0	+++	+	0	0	0	16, 17
MobQues47		++	?	0	0	0	0	0	0	0	18
TUG		++	?	0	+++	0	++	0	?	?	13, 15, 19
CHQ		±	-	+	0	±	±	0	0	0	20
PedsQL		±	±	+	0	++	±	0	0	?	20
CPQoL		+	0	?	0	?	+	0	0	0	21
GOAL		+	0	+	?	0	+++	0	0	±	*
PROMIS		0	0	+	0	0	+	+	0	+	*
PODCI		±	0	+++	0	0	+++	0	?	±	*

* There are no existing COSMIN reviews available, and researchers have assessed the quality of the evidence.

Table 3 Feasibility criteria of the shortlisted outcome measures.

Outcome Measure		Administration format	Age	Administration time	Length of survey	Equipment needed	Assessor training	Cost	References
Gait Analysis	GGI	Clinician	Not specified	Not specified	16 items	Gait lab, software	Yes	Lab: Yes	22
	GDI	Clinician	Not specified	Not specified	9 items	Gait lab, software	Yes	Lab: Yes	22
	GPS	Clinician	Not specified	Not specified	9 items	Gait lab, software	Yes	Lab: Yes	22
EVGS		Clinician	Not specified	Not specified	17 items	Video recordings	Yes	Software: Yes	23-25
OGS		Clinician	Not specified	Not specified	Not specified	Video recordings	Yes	Free	23, 24
PRS		Clinician	Not specified	Not specified	6 items	Video recordings	Yes	Free	23, 24
GMFM		Clinician	0-12 y	45-60 min	66 items	Computer, software	Yes	Manual: Yes	26, 27
FMS		Clinician	4-18 y	5-10 min	3 items	No	No	Free	15, 28
Gillette FAQ		Self- Proxy-reported	Not specified	5 min	22 items	No	No	Free	8, 24
PEDI		Proxy-reported	6m-8 y	30-60 min	197 items	Software	No	Yes	26, 29
GMPM		Clinician	5m-12 y	45-60 min	20 items	Computer, software	Yes	Yes	16
FIM		Clinician	7-18 y	30-45 min	18 items	No	No	Free	26, 30
MobQues47		Proxy-reported	2-18 y	15-20 min	47 items	No	No	Free	31
TUG		Clinician	3-18 y	5 min	5 items	Standard armchair, stopwatch	No	Free	15
CHQ		Self- Proxy-reported	Self-report: 10-18 y<br>Proxy-report: 5-18 y	Self-report: 16-25 min<br>Proxy-report: 5-15 min	Self-report: 87 items<br>Proxy-report: 28-50 items	Computer	Minimum training on scoring	Free	32, 33
PedsQL		Self- Proxy-reported	Self-report: 5-18 y<br>Proxy-report: 2-18 y	5-10 min	23 items	None	No	Yes	26, 34-36
CP-QoL		Self- Proxy-reported	Self-report: 9-12 y<br>Proxy-report: 4-12 y	15-25 min	Self-report = 53 items<br>Proxy-report = 66 items	No	No	Free	37, 38
GOAL		Proxy-reported	9-18 y	12-19 min	48 items	Form	No	Free	1, 39
PROMIS		Self- Proxy-reported	Self-report: 8-17 y<br>Proxy-report: 5-17 y	Not specified	Not specified	Computer	No	Free	26, 40-42
PODCI		Self- Proxy-reported	Self-report: 8-17 y<br>Proxy-report: 2-18 y	10-18 min	Self-report: 83 items (11–18 y)<br>Proxy-report: 86 items (11-18 y)<br>Proxy report: 86 items (2-10 y)	Computer	No	Free	26, 41

References

1. Thomason P, Tan A, Donnan A, Rodda J, Graham HK, Narayanan U. The Gait Outcomes Assessment List (GOAL): validation of a new assessment of gait function for children with cerebral palsy. *Dev Med Child Neurol*. Jun 2018;60(6):618-623. doi:doi:10.1111/dmcn.13722
2. Thomason P, Graham H, Narayanan U. The Gait Outcomes Assessment List (GOAL): responsiveness to change in gait function for children with cerebral palsy. 2011:
3. Karpati F, Weir S, Narayanan U. Gait Outcomes Assessment List (GOAL): Development of a patient priority-centered outcome measure for children with ambulatory cerebral palsy. University of Toronto; 2015. p. 1-2.
4. Boyer ER, Palmer M, Walt K, Georgiadis AG, Stout JL. Validation of the Gait Outcomes Assessment List questionnaire and caregiver priorities for individuals with cerebral palsy. *Dev Med Child Neurol*. Sep 17 2021;doi:10.1111/dmcn.15054
5. Damiano DL, Gilgannon MD, Abel MF. Responsiveness and uniqueness of the pediatric outcomes data collection instrument compared to the gross motor function measure for measuring orthopaedic and neurosurgical outcomes in cerebral palsy. *J Pediatr Orthop*. Sep-Oct 2005;25(5):641-5. doi:10.1097/01.bpo.0000167079.83835.22
6. Daltroy LH, Liang MH, Fossel AH, Goldberg MJ, The Pediatric Outcomes Instrument Development G. The POSNA Pediatric Musculoskeletal Functional Health Questionnaire: Report on Reliability, Validity, and Sensitivity to Change. *J Pediatr Orthop*. 1998;18(5)
7. McCarthy ML, Silberstein CE, Atkins EA, Harryman SE, Sponseller PD, Hadley-Miller NA. Comparing reliability and validity of pediatric instruments for measuring health and well-being of children with spastic cerebral palsy. *Dev Med Child Neurol*. Jul 2002;44(7):468-76. doi:10.1017/s0012162201002377
8. Pencharz J, Young NL, Owen JL, Wright JG. Comparison of Three Outcomes Instruments in Children. *J Pediatr Orthop*. 2001;21(4)
9. Vitale MG, Roye EA, Choe JC, Hyman JE, Lee FY, Roye DP, Jr. Assessment of Health Status in Patients With Cerebral Palsy: What is the Role of Quality-of-Life Measures? *J Pediatr Orthop*. 2005;25(6)
10. Kratz AL, Slavin MD, Mulcahey MJ, Jette AM, Tulsky DS, Haley SM. An examination of the PROMIS(®) pediatric instruments to assess mobility in children with cerebral palsy. *Qual Life Res*. Dec 2013;22(10):2865-76. doi:10.1007/s11136-013-0397-6
11. Coster WJ, Ni P, Slavin MD, et al. Differential item functioning in the Patient Reported Outcomes Measurement Information System Pediatric Short Forms in a sample of children and adolescents with cerebral palsy. *Dev Med Child Neurol*. Nov 2016;58(11):1132-1138. doi:10.1111/dmcn.13138
12. Mulcahey MJ, Haley SM, Slavin MD, et al. Ability of PROMIS Pediatric Measures to Detect Change in Children With Cerebral Palsy Undergoing Musculoskeletal Surgery. *J Pediatr Orthop*. Oct-Nov 2016;36(7):749-56. doi:10.1097/bpo.0000000000000533
13. Zanudin A, Mercer TH, Jagadamma KC, van der Linden ML. Psychometric properties of measures of gait quality and walking performance in young people with Cerebral Palsy: A systematic review. *Gait Posture*. Oct 2017;58:30-40. doi:doi:10.1016/j.gaitpost.2017.07.005
14. Debuse D, Brace H. Outcome measures of activity for children with cerebral palsy: a systematic review. *Pediatr Phys Ther*. Fall 2011;23(3):221-31. doi:10.1097/PEP.0b013e318227bbc6
15. Himuro N, Abe H, Nishibu H, Seino T, Mori M. Easy-to-use clinical measures of walking ability in children and adolescents with cerebral palsy: a systematic review. *Disabil Rehabil*. May 2017;39(10):957-968. doi:doi:10.1080/09638288.2016.1175036
16. Pavão SL, Silva FPS, Dusing SC, Rocha NACF. Saleh Clinical tools designed to assess motor abilities in children with cerebral palsy. *Dev Neurorehabil*. 2017/04/03 2017;20(3):149-159. doi:10.3109/17518423.2016.1150359
17. Ferre-Fernández M, Murcia-González MA, Barnuevo Espinosa MD, Ríos-Díaz J. Measures of Motor and Functional Skills for Children With Cerebral Palsy: A Systematic Review. *Pediatr Phys Ther*. 2020;32(1):12-25.
18. Ammann-Reiffer C, Bastiaenen CHG, de Bie RA, van Hedel HJA. Measurement Properties of Gait-Related Outcomes in Youth With Neuromuscular Diagnoses: A Systematic Review. *Phys Ther*. 2014;94(8):1067-1082. doi:10.2522/ptj.20130299
19. Saether R, Helbostad JL, Riphagen, II, Vik T. Clinical tools to assess balance in children and adults with cerebral palsy: a systematic review. *Dev Med Child Neurol*. Nov 2013;55(11):988-99. doi:10.1111/dmcn.12162
20. Janssens A, Rogers M, Thompson Coon J, et al. A systematic review of generic multidimensional patient-reported outcome measures for children, part II: evaluation of psychometric performance of English-language versions in a general population. *Value Health*. Mar 2015;18(2):334-45. doi:10.1016/j.jval.2015.01.004
21. Coombes LH, Wiseman T, Lucas G, Sangha A, Murtagh FEM. Health-related quality-of-life outcome measures in paediatric palliative care: A systematic review of psychometric properties and feasibility of use. *Palliat Med*. 2016/12/01 2016;30(10):935-949. doi:10.1177/0269216316649155

22. Danino B, Erel S, Kfir M, et al. Are Gait Indices Sensitive Enough to Reflect the Effect of Ankle Foot Orthosis on Gait Impairment in Cerebral Palsy Diplegic Patients? *J Pediatr Orthop*. Apr-May 2016;36(3):294-8. doi:10.1097/bpo.0000000000000429
23. Rathinam C, Bateman A, Peirson J, Skinner J. Observational gait assessment tools in paediatrics--a systematic review. *Gait Posture*. Jun 2014;40(2):279-85. doi:10.1016/j.gaitpost.2014.04.187
24. Harvey A, Gorter JW. Video gait analysis for ambulatory children with cerebral palsy: Why, when, where and how! *Gait Posture*. 2011/03/01/ 2011;33(3):501-503. doi:https://doi.org/10.1016/j.gaitpost.2010.11.025
25. Tzikalagia T, Ramdharry G. Using the Edinburgh Visual Gait Score to assess gait in children with cerebral palsy: A feasibility evaluation. *Int J Ther Rehabil*. 2017;24(10):419-426. doi:10.12968/ijtr.2017.24.10.419
26. Schiariti V, Klassen AF, Cieza A, et al. Comparing contents of outcome measures in cerebral palsy using the International Classification of Functioning (ICF-CY): a systematic review. *Eur J Paediatr Neurol*. Jan 2014;18(1):1-12. doi:doi:10.1016/j.ejpn.2013.08.001
27. Gross Motor Function Measure (GMFM). CanChild. Accessed 17 Febraury, 2020. https://canchild.ca/en/resources/44-gross-motor-function-measure-gmfm
28. Graham HK, Harvey A, Rodda J, Nattrass GR, Pirpiris M. The functional mobility scale (FMS). *J Pediatr Orthop*. 2004;24(5):514-520.
29. Haley SM, New England Medical Center Hospital PRG. *Pediatric evaluation of disability inventory (PEDI): development, standardization and administration manual (Kit)*. New England Medical Center Hospital, PEDI Research Group; 1992.
30. James S, Ziviani J, Boyd R. A systematic review of activities of daily living measures for children and adolescents with cerebral palsy. *Dev Med Child Neurol*. Mar 2014;56(3):233-44. doi:10.1111/dmcn.12226
31. Roorda LD, Scholtes VA, van der Lee JH, Becher J, Dallmeijer AJ. Measuring Mobility Limitations in Children With Cerebral Palsy: Development, Scalability, Unidimensionality, and Internal Consistency of the Mobility Questionnaire, MobQues47. *Arch Phys Med Rehabil*. 2010;91(8):1194-1209. doi:10.1016/j.apmr.2010.05.009
32. Hullmann SE, Ryan JL, Ramsey RR, Chaney JM, Mullins LL. Measures of general pediatric quality of life: Child Health Questionnaire (CHQ), DISABKIDS Chronic Generic Measure (DCGM), KINDL-R, Pediatric Quality of Life Inventory (PedsQL) 4.0 Generic Core Scales, and Quality of My Life Questionnaire (QoML). *Arthritis Care Res*. 2011;63(S11):S420-S430. doi:10.1002/acr.20637
33. Schiariti V, Fayed N, Cieza A, Klassen A, O'Donnell M. Content comparison of health-related quality of life measures for cerebral palsy based on the International Classification of Functioning. *Disabil Rehabil*. 2011;33(15-16):1330-9. doi:doi:10.3109/09638288.2010.531371
34. Narayanan UG. Chapter 8 - Outcomes Assessment of Fractures in Children. In: Mencio GA, Swiontkowski MF, eds. *Green's Skeletal Trauma in Children*. W.B. Saunders; 2015:130-141.
35. Janssens A, Thompson Coon J, Rogers M, et al. A systematic review of generic multidimensional patient-reported outcome measures for children, part I: descriptive characteristics. *Value Health*. Mar 2015;18(2):315-33. doi:10.1016/j.jval.2014.12.006
36. Janssens L, Gorter JW, Ketelaar M, Kramer WLM, Holtslag HR. Health-related quality-of-life measures for long-term follow-up in children after major trauma. *Qual Life Res*. 2008;17(5):701-713. doi:10.1007/s11136-008-9339-0
37. The Cerebral Palsy Quality of Life Questionnaire. Australasian Academy of Cerebral Palsy and Developmental Medicine. Accessed 17 February, 2020. https://www.ausacpdm.org.au/research/cpqol/
38. Carlon S, Shields N, Yong K, Gilmore R, Sakzewski L, Boyd R. A systematic review of the psychometric properties of Quality of Life measures for school aged children with cerebral palsy. *BMC Pediatr*. 2010;10(81):1-11. doi:10.1186/1471-2431-10-81
39. Dermott JA. *Development and Sensibility Evaluation of the Gait Outcomes Assessment List (GOAL) Module for Children with Lower Limb Anomalies* University of Toronto; 2015.
40. Allen DD, Gorton GE, Oeffinger DJ, Tylkowski C, Tucker CA, Haley SM. Analysis of the pediatric outcomes data collection instrument in ambulatory children with cerebral palsy using confirmatory factor analysis and item response theory methods. *J Pediatr Orthop*. 2008;28(2):192-198. doi:10.1097/BPO.0b013e3181652185
41. Klepper SE. Measures of pediatric function: Child Health Assessment Questionnaire (C-HAQ), Juvenile Arthritis Functional Assessment Scale (JAFAS), Pediatric Outcomes Data Collection Instrument (PODCI), and Activities Scale for Kids (ASK). *Arthritis Care Res*. 2011;63(S11):S371-S382. doi:10.1002/acr.20635
42. Health Measures. Introduction to PROMIS. Accessed 07 Febraury, 2020. http://www.healthmeasures.net/explore-measurement-systems/promis/intro-to-promis
