# Supplementary material for: Development of a set of core outcome measures for ambulant children with cerebral palsy after lower limb orthopaedic surgery
Source: Dev Med Child Neurol. 2025 Dec 29;68(8):1127–38. doi: 10.1111/dmcn.70133 (PMC13340623; doi:10.1111/dmcn.70133)
Supplement: Supplementary file 5 — Appendix S5: Updated search (August 2020–July 2024). [file DMCN-68-1127-s004.pdf]

Updated Search (August 2020 to July 2024)

COSMIN Database

Updated search on the COSMIN database search identified further 11 reviews. The titles and abstracts of these reviews were extracted and screened. Of these, 10 were excluded: 2 did not provide data regarding relevant outcome measures, 4 were out of the current review scope, 1 was structured/ scoping reviews of literature, and 3 reviews did not specify the health conditions that had been involved in the studies (e.g., children with neurological disorders). This process left only one review for inclusion (Table 1).

Table 1 Reviews identified from COSMIN database

Author/ year	Title	Condition	Review Focus	Outcome measures	Ref
Smith et al (2022)	Pain coping tools for children and young adults with a neurodevelopmental disability: A systematic review of measurement properties	Neurodevelopmental disability	Pain	CP-QoL	1

PubMed Database

Updated search on the PubMed search identified further 50 studies. The titles and abstracts of these studies were screened. Of these, 46 were excluded as the studies did not provide data regarding the psychometric properties of the outcome measures. Only four studies were included and added to the original search (Table 2).

Table 2 Characteristics of the included studies and ratings of methodological quality and quality of evidence

Outcome measure	Study	n, age (y)	Psychometric properties	Results	(COSMIN) rating	Quality of Evidence	Ref
GOAL	Bonfert et al (2022)		Construct validity	In three domains, caregivers rated their children significantly lower than children rated themselves	Good	+	2
	Stout et al (2024)	112, 4-17	Reliability	SEM: 3.1 points for the cohort<br>GMFCS level I = 2.3<br>GMFCS level II = 3.8<br>GMFCS level III = 3.6<br>ICC for the total score = 0.96 (95% CI=0.94–0.97)<br>ICC for individual domains = 0.73 to 0.96<br>Cohen's quadratic weighted kappas (0.35–0.90)<br>Linear weighted kappas (0.39–0.76)	Excellent	+	3
	Munger et al (2024)	622, 11:2	Internal consistency	Cronbach alphas (≥0.80)	Excellent	+	4
	Nordbye-Nielsen et al (2024)*	59, 5-18	Reliability	Child: ICC (95%CI) = 0.91 (0.83; 0.96)<br>SEM= 4.02<br>Parent: ICC (95%CI) = 0.83 (0.67;0.91)<br>SEM= 4.79	Good	+	5

	Discriminative validity	The children's scores were significantly different between the GMFCS levels for the total GOAL score (p < 0.01)<br>The parents' total GOAL score was higher if their children had GMFCS level I than level II, but this difference did not reach statistical significance (p = 0.07)	Good	+
	Concurrent validity	Challenge-20:<br>Child: Spearman's rho (0.30, 95% CI −0.01 to 0.56, p = 0.06)<br>Parent: Spearman's rho (0.34, 95% CI 0.04–0.59, p = 0.03)	Good	-
	Face/ Content validity	All GOAL domains and items were considered acceptable and relevant to most children, parents and professionals.	Excellent	+

GOAL: Gait Outcome Assessment List, PODCI: Pediatric Outcomes Data Collection Instrument PROMIS: Patient-Reported Outcomes Measurement Information System.
* Other language

References

1. Smith NL, Smith MG, Gibson N, Imms C, Thornton Al, Harvey AR. Pain coping tools for children and young adults with a neurodevelopmental disability: A systematic review of measurement properties. *Developmental Medicine & Child Neurology*. 2023;65(3):318-328. doi:https://doi.org/10.1111/dmcn.15410
2. Bonfert MV, Jelesch E, Hartmann J, et al. Test-Retest Reliability and Construct Validity of the German Translation of the Gait Outcome Assessment List (GOAL) Questionnaire for Children with Ambulatory Cerebral Palsy. *Neuropediatrics*. Apr 2022;53(2):96-101. doi:10.1055/s-0040-1722688
3. Stout JL, Thill M, Munger ME, Walt K, Boyer ER. Reliability of the Gait Outcomes Assessment List questionnaire. *Dev Med Child Neurol*. Jan 2024;66(1):61-69. doi:10.1111/dmcn.15677
4. Munger ME, Shippee ND, Beebe TJ, Novacheck TF, Virnig BA. Factor analysis of the Gait Outcomes Assessment List's goal questions: A new method to measure goal prioritization in ambulatory individuals with cerebral palsy. *Dev Med Child Neurol*. Jan 2024;66(1):70-81. doi:10.1111/dmcn.15704
5. Nordbye-Nielsen K, Maribo T, Rahbek O, Narayanan U, Møller-Madsen B. The Danish child and parent Gait Outcomes Assessment List questionnaires were reliable and valid for cerebral palsy. *Acta Paediatr*. Feb 2024;113(2):353-361. doi:10.1111/apa.17046
