# Supplementary figures and images for: Development of a set of core outcome measures for ambulant children with cerebral palsy after lower limb orthopaedic surgery
Source: Dev Med Child Neurol. 2025 Dec 29;68(8):1127–38. doi: 10.1111/dmcn.70133 (PMC13340623; doi:10.1111/dmcn.70133)

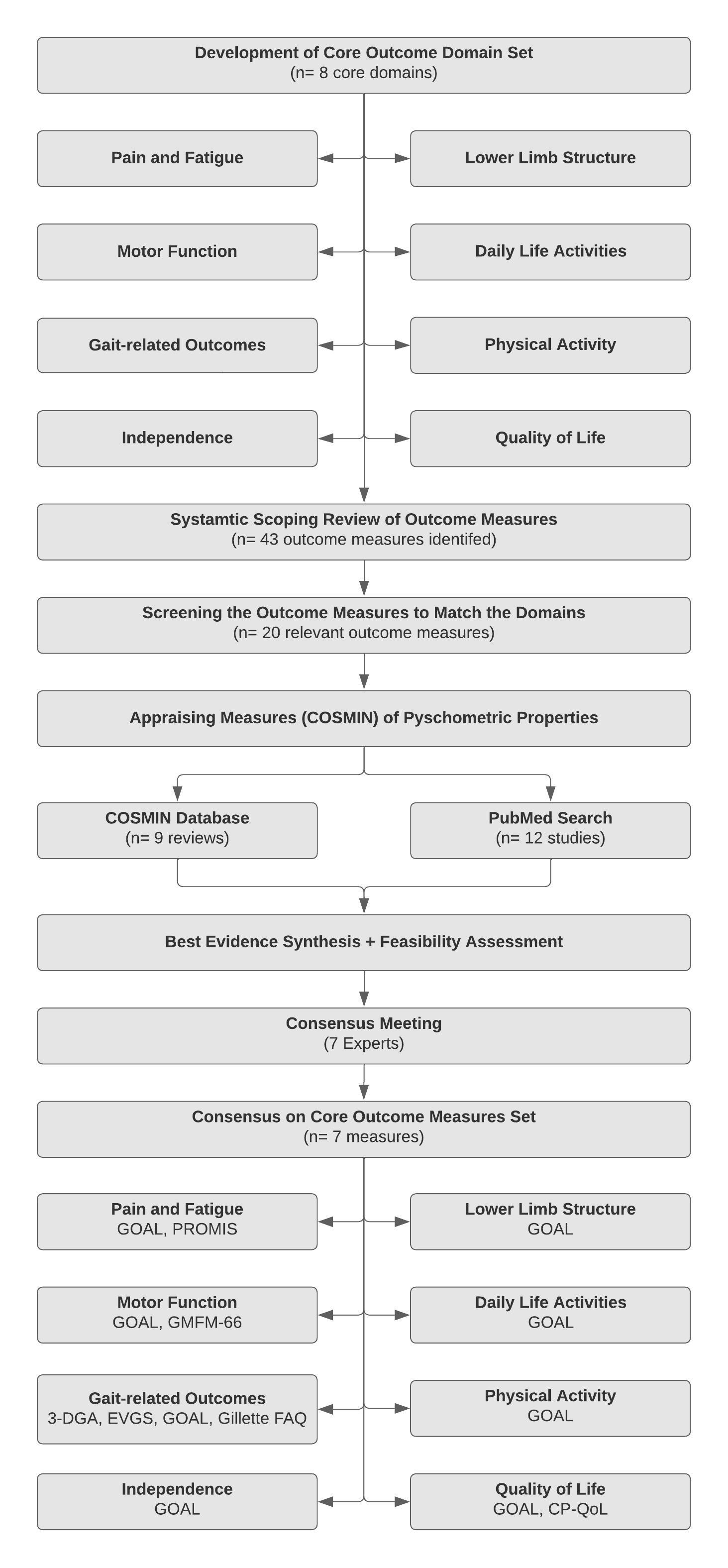

Supplement: Supplementary file 6 — Figure S1: Flowchart of the development of the core outcome measures set. [file DMCN-68-1127-s003.jpeg]
